# Long noncoding RNA LINC00336 inhibits ferroptosis in lung cancer by functioning as a competing endogenous RNA

**DOI:** 10.1038/s41418-019-0304-y

**Published:** 2019-02-20

**Authors:** Min Wang, Chao Mao, Lianlian Ouyang, Yating Liu, Weiwei Lai, Na Liu, Ying Shi, Ling Chen, Desheng Xiao, Fenglei Yu, Xiang Wang, Hu Zhou, Ya Cao, Shuang Liu, Qin Yan, Yongguang Tao, Bin Zhang

**Affiliations:** 1 Key Laboratory of Carcinogenesis and Cancer Invasion, Ministry of Education, Department of Pathology, Xiangya Hospital, Central South University, Changsha, Hunan 410078 China; 2 Department of Histology and Embryology, School of Basic Medicine, Central South University, Changsha, Hunan 410013 China; 3 NHC Key Laboratory of Carcinogenesis (Central South University), Cancer Research Institute, Central South University, Changsha, Hunan 410078 China; 4 Department of Oncology, Institute of Medical Sciences, Xiangya Hospital, Central South University, Changsha, Hunan 410008 China; 5 Department of Pathology, Xiangya Hospital, Central South University, Changsha, Hunan 410008 China; 6 Department of Thoracic Surgery, Second Xiangya Hospital, Central South University, Changsha, 410011 China; 7 Shanghai Institute of Material Medica, Chinese Academy of Sciences (CAS), 555 Zu Chongzhi Road, Zhangjiang Hi-Tech Park, Shanghai, 201203 China; 8 Department of Pathology, Yale School of Medicine, New Haven, CT 06520 USA

**Keywords:** Non-small-cell lung cancer, Tumour biomarkers

## Abstract

The regulatory loop between long noncoding RNAs (lncRNAs) and microRNAs has a dynamic role in transcriptional and translational regulation, and is involved in cancer. However, the regulatory circuitry between lncRNAs and microRNAs in tumorigenesis remains elusive. Here we demonstrate that a nuclear lncRNA LINC00336 is upregulated in lung cancer and functions as an oncogene by acting as a competing endogenous RNA (ceRNAs). LINC00336 bound RNA-binding protein ELAVL1 (ELAV-like RNA-binding protein 1) using nucleotides 1901–2107 of LINC00336 and the RRM interaction domain and key amino acids (aa) of ELAVL1 (aa 101–213), inhibiting ferroptosis. Moreover, ELAVL1 increased LINC00336 expression by stabilizing its posttranscriptional level, whereas LSH (lymphoid-specific helicase) increased ELAVL1 expression through the p53 signaling pathway, further supporting the hypothesis that LSH promotes LINC00336 expression. Interestingly, LINC00336 served as an endogenous sponge of microRNA 6852 (MIR6852) to regulate the expression of cystathionine-β-synthase (CBS), a surrogate marker of ferroptosis. Finally, we found that MIR6852 inhibited cell growth by promoting ferroptosis. These data show that the network of lncRNA and ceRNA has an important role in tumorigenesis and ferroptosis.

## Introduction

Lung cancer is the leading cause of cancer-related deaths and is the most common cancer worldwide and in China, resulting in more than 2.8 million deaths in 2015, ~ 40% of which involved lung adenocarcinomas (ADCs) [1]. Lung cancer is divided into small cell lung cancer and non-small cell lung cancer (NSCLC), including ADC, and squamous cell carcinoma (SCC), which account for 80–85% of all lung cancer cases, respectively [2]. Epigenetic modifiers, including long noncoding RNAs (lncRNAs) and microRNAs (miRNAs), have important roles in the development and progression of NSCLC [2, 3]. Thus, a great challenge lies ahead in understanding the molecular mechanisms of lung cancer in identifying novel prognostic molecular markers that can facilitate the development of appropriate therapeutic strategies earlier in the development of this cancer.

lncRNAs have attracted significant attention because of their emerging role in cancer [4, 5]. Oncogenes and cancer epigenetic discoveries over the past decade have revealed that numerous epigenetic modifiers such as lncRNAs are involved in the progression of various cancers [6–9], whereas tens of thousands of lncRNAs may be encoded in the human genome [10, 11]. Moreover, lncRNAs have been implicated in a large number of transcription regulatory processes such as sponging activity for miRNAs [12–14]. Sponging activity indicates that coding and noncoding RNAs may be a component of common regulatory circuitries in which they control one another through their ability to compete for miRNA binding, supporting the term “competing endogenous RNA” (ceRNA) [15]. Clearly, the precise role of many coding and noncoding ceRNAs remains elusive.

Lymphoid-specific helicase (LSH), a member of the SNF2 family of chromatin-remodeling ATPases, is critical to normal development, because it establishes correct DNA methylation levels and patterns [16–19]. LSH maintains genome stability in mammalian somatic cells [20, 21]. LSH also contributes to the malignant progression of prostate cancer, melanoma, nasopharyngeal carcinoma, and glioma [22–27]. Our recent reports indicate that the lncRNA HOTAIR interacts with LSH; in turn, the intact complex affects target genes [28]. However, the regulation interplay of lncRNAs and LSH remains unknown.

The reprogramming of cellular metabolism, including the regulation of apoptotic and necrotic cell death, is necessary for tumorigenesis [29–31]. Ferroptosis, a newly discovered mode of nonapoptotic cell death, involves metabolic dysfunction that results in intracellular metabolic process glutaminolysis and the production of iron-dependent reactive oxygen species (ROS), the iron carrier protein transferrin, and mitochondrial superoxide, as well as membrane potential decrease and the formation of other related regulators such as p53 [32–36]. Iron and iron derivatives are essential for the functioning of ROS-producing enzymes, suggesting that iron might function as a trigger or mediator of cell death signaling, particularly in ferroptosis [32, 37], further indicating that iron-containing enzymes are essential for ferroptosis. Herein, we investigate LINC00336-associated epigenetic regulation in ferroptosis and tumorigenicity in lung cancer.

## Results

### LSH promotes the expression of LINC00336 in lung cancer

**Fig. 1:**
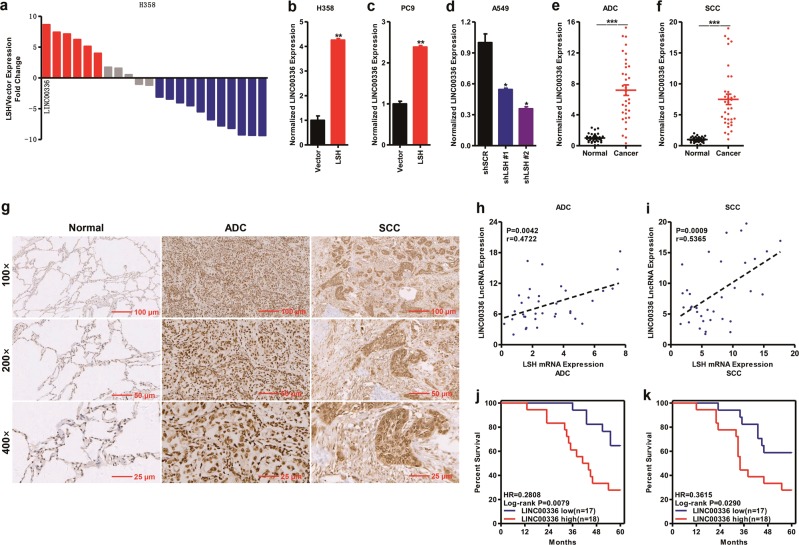
LINC00336 is an LSH-regulated lncRNA in lung cancer and is closely related to patient prognosis. **a** LncRNA gene ranking correlated with LSH expression was assessed by RNA-sequencing. **b**, **c** The RNA level of LINC00336 increased after stably expressing LSH in H358 and PC9 cells. **d** The RNA level of LINC00336 decreased after the knockdown of LSH in A549 cells. **e**, **f** qRT-PCR shows an increased expression of LINC00336 in 70 paired lung cancer relative to corresponding normal lung tissue samples. **e** ADC. **f** SCC. **g** In situ hybridization was used to analyze increased LINC00336 expression levels in lung cancer. **h**,**i** Correlations between LSH mRNA and LINC00336 LncRNA levels in lung ADC (**h**) and SCC (**i**) were analyzed. The *r*-values and *P*-values were derived via Pearson’s correlation analysis. **j**, **k** Kaplan–Meier curves for overall survival rates associated with LINC00336 expression in ADC (**j**) and SCC (**k**). Data are shown as the mean ± SEM; *n* ≥ 3 independent experiments, two-tailed Student’s *t*-test: ns nonsignificant (*p* > 0.05), *P < 0.05, **P < 0.01, ***P < 0.001

To validate lncRNAs that might be regulated by LSH, LSH expression levels were detected in lung cancer cell lines, and were overexpressed in H358 and PC9 cell lines and knocked down in A549 cell line by using lentivirus. We selected 22 differentially expressed lncRNAs from RNA-sequencing data and analyzed their expression in H358 cells with stable LSH expression [38]. The quantitative reverse transcription PCR (qRT-PCR) analysis confirms that LSH aberrantly regulates a set of lncRNAs in H358 cells. We focused mainly on upregulated lncRNA (LINC00336) as top-ranked candidates in lung cancer as indicated by a significant *P*-value (*P* = 0.0002) (Fig. 1a). Next, we confirmed that LINC00336 was upregulated after the overexpression of LSH in H358 and PC9 cell lines, whereas the knockdown of LSH in A549 cells decreased LINC00336 expression levels when using two separate short hairpin RNA (shRNA) sequences (Fig. 1b–d and Supplementary Fig. 1a–d). We then assessed levels of LINC00336 in an independent panel of 70 primary lung tumors and normal lung tissues. We found that LINC00336 was upregulated in both lung ADC and SCC primary tumors (Fig. 1e, f). In situ hybridization results showed that LINC00336 was localized primarily in the nucleus of lung cancer tissues, and that LINC00336 levels significantly increased in both lung ADC and SCC (Fig. 1g and Supplementary Fig. 1e, f). Furthermore, the nuclear localization of LINC00336 was confirmed by subcellular fractionation analyses of H358, SPC-A-1, PC9, and A549 cells (Supplementary Fig. 1g-j), suggesting that LINC00336 may perform its biological functions in the nucleus. A strong correlation between LSH and LINC00336 expression was found in lung ADC and SCC, and we also confirmed via western blotting that LSH was highly expressed in lung ADC and SCC primary tumors (Fig. 1h, i and Supplementary Fig. 1k). Finally, we found patients who had relatively higher levels of LINC00336 are associated with poor overall survival from lung cancers; this was assessed through a Kaplan–Meier analysis of a cohort of patients with lung cancer (Fig. 1j, k), indicating that LINC00336 might be used as a biomarker of NSCLC.

### Overexpression of LINC00336 promotes cell growth, colony formation, tumor formation, and inhibits ferroptosis

**Fig. 2:**
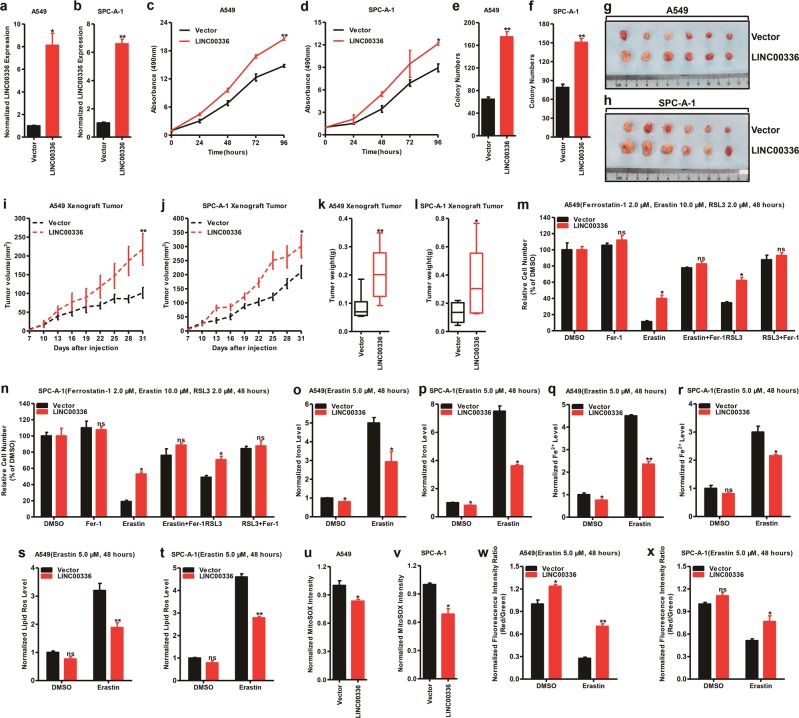
The overexpression of LINC00336 promotes cell growth, colony formation, tumor formation, and inhibits ferroptosis. **a**,**b** A qRT-PCR analysis was conducted to detect the level of LINC00336 in A549 (**a**) and SPC-A-1 (**b**) cells stably overexpressing LINC00336. **c**,**d** MTT assay was used to assess cell viability in A549 (**c**) and SPC-A-1 (**d**) cells stably overexpressing LINC00336. **e**,**f** Colony-formation assay of A549 (**e**) and SPC-A-1 (**f**) cells stably overexpressing LINC00336. **g**–**l** Nude mouse cells after the injection of A549 and SPC-A-1 stably expressing the control vector or LINC00336 expression plasmids are shown. Tumor formation was monitored at the indicated times (**i**,**j**), images (**g**,**h**) and weights (**k**,**l**) were recorded (*n* = 6). **m**,**n** MTT assays were used to analyze the responses of A549 (**m**) and SPC-A-1 (**n**) cells stably overexpressing LINC00336 to ferrostatin (2.0 μM), erastin (10.0 μM), and RSL3 (2.0 μM). **o**–**x** Levels of total iron (**o**,**p**), ferrous iron (**q**,**r**), lipid ROS (**s**,**t**), mitochondrial superoxide (**u**,**v**) and mitochondrial membrane potential (**w**,**x**) were analyzed in A549 and SPC-A-1 cells stably overexpressing LINC00336. Data are shown as the mean ± SEM; *n* ≥ 3 independent experiments, two-tailed Student’s *t*-test: ns nonsignificant (*p* > 0.05), *P < 0.05, **P < 0.01, ***P < 0.001

According to Kaplan–Meier analysis, the LINC00336 expression has more significant effect on the prognosis of patients with ADC (*P* = 0.0079) than SCC (*P* = 0.0290); thus, we chose ADC cell lines as research models. We detected the expression levels of LINC00336 in a panel of lung ADC cells; LINC00336 had low expression level in A549 and SPC-A-1 cell lines but was highly expressed in PC9 cell lines. Next, we used A549 and SPC-A-1 cell lines to overexpress LINC00336 and knock down LINC00336 in PC9 cell lines by using lentivirus. The efficiency of overexpression and knockdown was detected by qRT-PCR (Supplementary Fig. 2a and Fig. 2a, b). The overexpression of LINC00336 enhanced the growth ability of A549 and SPC-A-1 in vitro (Fig. 2c, d). Moreover, the overexpression of LINC00336 in A549 and SPC-A-1 cell lines significantly enhanced colony formation (Fig. 2e, f and Supplementary Fig. 2b). To address whether LINC00336 also has a role in lung cancer in vivo, we used a xenograft model. The injection of A549-LINC00336 and SPC-A-1-LINC00336 showed that LINC00336 overexpression significantly increased tumor sizes, volumes, and weights after 1 month of growth compared with A549-Vector and SPC-A-1-Vector cells (Fig. 2g–l), whereas the whole-body weight remained unchanged (Supplementary Fig. 2c, d). Together, our results demonstrate that LINC00336 overexpression is linked to cell growth, colonization, and tumor growth, suggesting that LINC00336 performs a critical oncogenic function in cancer progression.

Our previous studies indicated that LSH inhibits ferroptosis, a novel ferroptotic mode of nonapoptotic cell death, by decreasing intracellular levels of iron and lipid ROS [32, 34, 38]. We first treated cells using erastin or RSL3, inducers of ferroptosis, and we observed that LINC00336 overexpression limited the erastin-induced or RSL3-induced growth inhibition of A549 and SPC-A-1 cells (Fig. 2m, n). The overexpression of LINC00336 in A549 or SPC-A-1 cells can resist erastin-induced ferroptosis in a dose- and time-dependent manner (Supplementary Fig. 2e–h). Trypan blue staining results show that the percentage of dead cell decreased after the overexpression of LINC00336 (Supplementary Fig. 2i, j). Furthermore, we found that the overexpression of LINC00336 decreased intracellular concentrations of iron and Fe^2+^ (Fig. 2o–r). The overexpression of LINC00336 also decreased intracellular concentrations of lipid ROS and mitochondrial superoxide, and increased mitochondrial membrane potential in A549 and SPC-A-1 cells (Fig. 2s–x), indicating that the overexpression of LINC00336 induced resistance to ferroptosis.

### Knockdown of LINC00336 attenuates cell growth, colony formation, tumor formation, and promotes ferroptosis

**Fig. 3:**
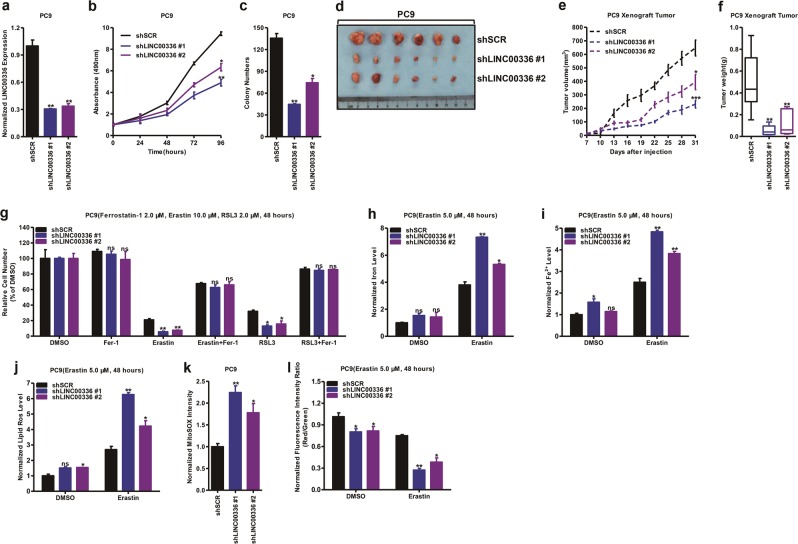
The knockdown of LINC00336 inhibits cell growth, colony formation, and tumor formation, and promotes ferroptosis. **a** qRT-PCR analysis was conducted to determine the level of LINC00336 in PC9 cells stably transfected with two distinct target shRNA vectors and control cells (shSCR). **b** An MTT assay was used to assess cell viability in PC9 cells after the knockdown of LINC00336. **c** Growth in the plate was measured with a colony formation assay of PC9 cells after the knockdown of LINC00336. **d**–**f** Nude mice are shown after the injection of PC9 cell knockdown LINC00336. Tumor formation (**e**) was monitored at the indicated times; images (**d**) and weights (**f**) were recorded (*n* = 6). **g** MTT assays were used to analyze the responses of PC9 cells subjected to the stable knockdown of LINC00336 to ferrostatin (2.0 μM), erastin (10.0 μM), and RSL3 (2.0 μM). **h**–**l** Levels of total iron (**h**), ferrous iron (**i**), lipid ROS (**j**), and mitochondrial superoxide (**k**), and mitochondrial membrane potential (**l**) were analyzed in PC9 cells subjected to the stable knockdown of LINC00336. Data are shown as the mean ± SEM; *n* ≥ 3 independent experiments, two-tailed Student’s *t*-test: ns nonsignificant (*p* > 0.05), *P < 0.05, **P < 0.01, ***P < 0.001

To further uncover the physiological role of LINC00336 in lung cancer, we performed the stable knockdown of LINC00336 in PC9 cells. Using two separate sequences, the knockdown approach successfully reduced RNA levels of LINC00336 (Fig. 3a). The knockdown of LINC00336 in PC9 cells significantly slowed cell growth (Fig. 3b). Moreover, the knockdown of LINC00336 significantly inhibited colony formation (Fig. 3c and Supplementary Fig. 3a). To address whether LINC00336 has a role in lung cancer in vivo, we used a xenograft model. We injected PC9 cells into nude mice and observed that LINC00336 depletion significantly impaired tumor size, volume, and weight (Fig. 3d–f); however, body weights did not change significantly in either group (Supplementary Fig. 3b).

Finally, we observed that the knockdown of LINC00336 increased the erastin-induced or RSL3-induced growth inhibition of PC9 cells (Fig. 3g). PC9 cells became more sensitive to erastin-induced ferroptosis in a dose- and time-dependent manner with the knockdown of LINC00336 (Supplementary Fig. 3c, d). The trypan blue staining results show that the percentage of dead cells present increased after the knockdown of LINC00336 (Supplementary Fig. 3e). We found that decreased LINC00336 expression levels increased intracellular concentrations of iron and Fe^2+^ (Fig. 3h, i), whereas the knockdown of LINC00336 also increased intracellular concentrations of lipid ROS and mitochondrial superoxide, and decreased the mitochondrial membrane potential of PC9 cells (Fig. 3j–l). Together, our results demonstrate that LINC00336 is linked to cell growth, colonization, tumor growth, and ferroptosis, suggesting that LINC00336 performs a critical oncogenic function in cancer progression.

### RNA pulldowns identify ELAVL1 as an LINC00336-interacting protein

**Fig. 4:**
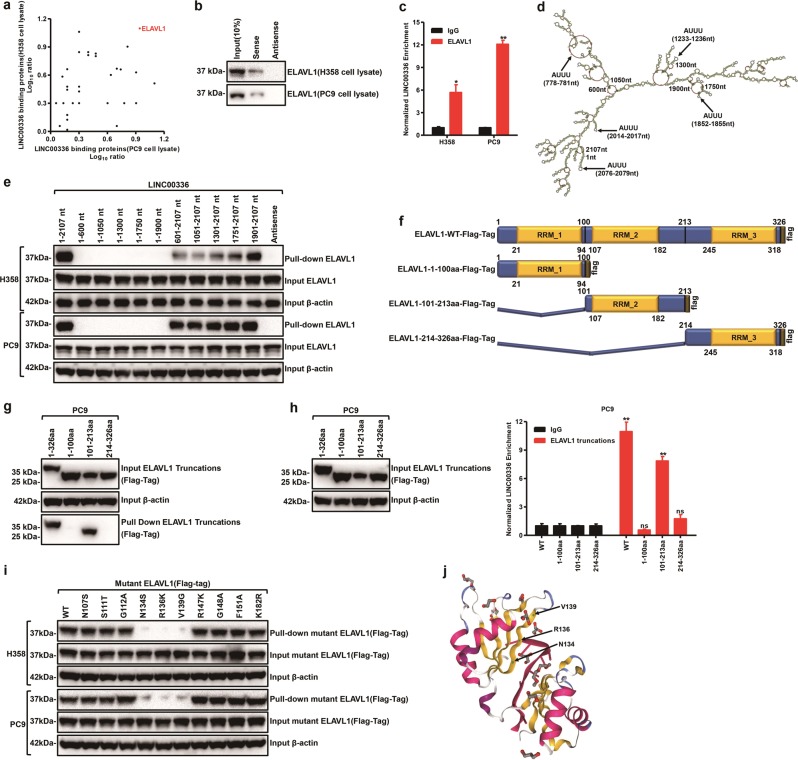
LINC00336 interacts with RNA-binding protein ELAVL1. **a** The LINC00336-interacting proteins were annotated with their Log^10^ ratio in H358 and PC9 cell lysate. **b** Immunoblot analysis of LINC00336-binding protein ELAVL1 in H358 and PC9 cells. **c** Levels of LINC00336 binding to ELAVL1 relative to control IgG derived from three experiments conducted on H358 and PC9 cells. **d** The predicted secondary structure of LINC00336. **e** RNA pull-down analysis of ELAVL1 pulldown by in vitro-transcribed biotinylated LINC00336 (wild-type vs. domain truncation mutants). **f** The domain structure of ELAVL1 and the three Flag-tag ELAVL1 constructs used for mapping. **g** IB analysis of FLAG-tagged ELAVL1 truncations retrieved by biotinylated LINC00336. **h** RIP assays show the association between the ELAVL1 RRM domain and LINC00336. **i** N134S, R136K, and V139G mutants of ELAVL1 impair binding with LINC00336. **j** Computational modeling results show multiple binding sites for LINC00336 on ELAVL1. The green region is the binding site for LINC00336. Data are shown as the mean ± SEM; *n* ≥ 3 independent experiments, two-tailed Student’s *t*-test: ns nonsignificant (*p* > 0.05), *P < 0.05, **P < 0.01, ***P < 0.001

LncRNAs function through RNA-interacting proteins that regulate gene expression through various mechanisms [9, 39, 40]. We investigated whether LINC00336 may interact with certain cellular proteins to regulate biological functions. Using RNA pulldown assays, we identified a LINC00336–protein complex. The antisense strand of LINC00336 was used as a negative control. The mass spectrometry analysis results show that LINC00336 binds numerous proteins in H358 and PC9 cells; among these proteins, we were particularly interested in ELAVL1 (Fig. 4a). In addition, the RNA pull-down assay using biotin-LINC00336 and the antisense LINC00336 serving as a negative control confirm that LINC00336 interacted with ELAVL1 at the endogenous level in H358 and PC9 cells (Fig. 4b). RNA immunoprecipitation (RIP) assays using anti-ELAVL1 antibody confirm that ELAVL1 interacted with LINC00336 in H358 and PC9 cells (Fig. 4c). Taken together, ELAVL1 was confirmed to serve as a novel binding partner of LINC00336.

To determine which specific region(s) within LINC00336 contribute(s) to ELAVL1 binding, we predicted the secondary structure of LINC00336 using RNAfold Webserve (http://rna.tbi.univie.ac.at/cgi-bin/RNAfold.cgi) (Fig. 4d). We constructed ten different deletion fragments of LINC00336 and the 1901–2107-nucleotide (nt)-long region of LINC00336 with two ELAVL1-specific binding sequences (AUUU) bound to ELAVL1 as efficiently as the full-length LINC00336 in H358 and PC9 cells (Fig. 4e). To determine which specific region(s) within ELAVL1 contribute to LINC00336 binding, we constructed three different deletion fragments of ELAVL1 (Fig. 4f). The RRM_2 motif (101–213 amino acids) of ELAVL1 bound to LINC00336 with the same binding efficiency as full-length ELAVL1 (Fig. 4g). Using a RIP assay, we confirmed that LINC00336 bound to the RRM_2 motif (101–213 aa) of ELAVL1 (Fig. 4h). Taken together, our results indicate that 101–213 aa of ELAVL1 interacts with 1901–2107 nt of LINC00336.

To further establish key amino acid residues involved in the intact complex of ELAVL1 and LINC00336, we constructed ten single amino acids following computational predictions (Supplementary Fig. 4a). We found that mutants of ELAVL1 at N134S, R136K, and V139K completely attenuated the interaction between ELAVL1 and LINC00336, which is in agreement with the computational prediction (Fig. 4i, j). Taken together, the mutation sites of ELAVL1 as indicated are critical to the intact complex of the ELAVL1 and LINC00336 axis.

### ELAVL1 increases LINC00336 expression by stabilizing its posttranscriptional level

**Fig. 5:**
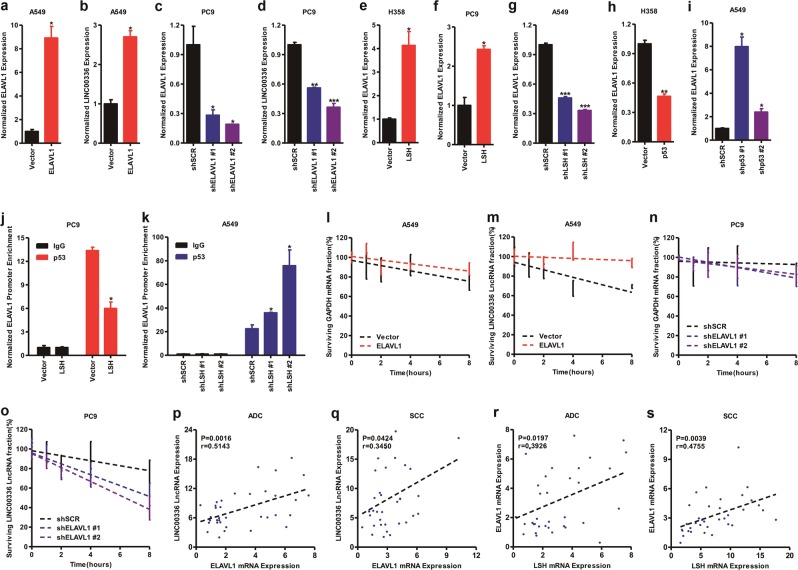
ELAVL1 stabilizes the LINC00336 RNA level. **a**, **b** The overexpression of ELAVL1 in A549 cells (**a**) increased LINC00336 expression levels (**b**). **c**, **d** The knockdown of ELAVL1 in PC9 cells (**c**) using two separate shRNA sequences and the LncRNA level of LINC00336 decreased (**d**). **e**–**g** mRNA levels of ELAVL1 increased in H358 and PC9 cells (**e**, **f**) stably overexpressing LSH and decreased after the knockdown of LSH in A549 cells (**g**). **h**, **i** mRNA levels of ELAVL1 decreased in H358 stably overexpressing p53 (**h**) and increased after the knockdown of p53 in A549 (**i**). **j**, **k** ChIP assays indicate that p53 is recruited to the promoter of ELAVL1 after the overexpression of LSH in PC9 (**j**) or the knockdown of LSH in A549 cells (**k**). **l**–**o** A549 overexpressed ELAVL1 cells (**l**, **m**) and PC9 knockdown ELAVL1 cells (**n**, **o**) were treated with actinomycin D (10 μg/ml) for the indicated times. LINC00336 and GAPDH mRNA stability was measured with RT-qPCR using β-actin as the loading control. **p**, **q** Correlations between ELAVL1 mRNA and LINC00336 LncRNA levels in lung ADC (**p**) and SCC (**q**) were analyzed. **r**, **s** Correlations between LSH and ELAVL1 mRNA levels in lung ADC (**r**) and SCC (**s**) were analyzed. *r*-values and *P*-values were derived via Pearson’s correlation analysis. Data are shown as the mean ± SEM; *n* ≥ 3 independent experiments, two-tailed Student’s *t*-test: ns nonsignificant (*p* > 0.05), *P < 0.05, **P < 0.01, ***P < 0.001

Given that ELAVL1 binds to many mRNAs, and that LINC00336 is highly expressed in lung cancer, we hypothesized that ELAVL1 may influence the posttranscriptional levels of interacting genes, including LINC00336. First, we detected ELAVL1 expression levels in H358, SPC-A-1, PC9, and A549 cells at mRNA and protein levels. ELAVL1 had low expression level in A549 cell line and was highly expressed in PC9 cell line (Supplementary Fig. 5a, b). We then detected ELAVL1 protein levels in ten lung cancer cases (five cases for ADC and five cases for SCC) and observed that ELAVL1 protein levels increased in lung cancer tissues (Supplementary Fig. 5c). We used A549 cell line to overexpressed ELAVL1 by lentivirus and found that ELAVL1 promoted LINC00336 RNA expression by using qRT-PCR (Fig. 5a, b). In addition, after the stable knockdown of ELAVL1 by using lentivirus in PC9 cells, LINC00336 expression was reduced (Fig. 5c, d), suggesting a regulatory role of ELAVL1 in LINC00336 expression.

Our previous findings derived from RNA-sequencing indicate that LSH regulates several genes, including *ELAVL1* [38]. LSH upregulated ELAVL1 after the stable overexpression of LSH in H358 and PC9 cells (Fig. 5e, f). Meanwhile, after the stable knockdown of LSH in A549 cells, ELAVL1 expression declined (Fig. 5g). Our recent findings show that p53 is sequestered in the nucleus by P53RRA, which is silenced by LSH [41], and we wondered whether p53 is involved in the regulation of ELAVL1. We observed that the overexpression of p53 attenuated ELAVL1 expression in H358 cells (Supplementary Fig. 5d and Fig. 5h), whereas the knockdown of p53 in A549 cells increased ELAVL1 expression levels (Supplementary Fig. 5e and Fig. 5i), indicating that p53 might regulate ELAVL1 expression. Next, using bioinformatics, we found that a putative binding site of p53 localizes upstream from ELAVL1 transcription start sites (TSS) (Supplementary Fig. 5f). Chromatin IP (ChIP) analysis results confirm that p53 was recruited to the promoter region of ELAVL1 and LSH attenuated the enrichment of p53 to the ELAVL1 promoter in PC9 cells (Fig. 5j). Furthermore, the depletion of LSH enhanced the recruitment of p53 binding to the ELAVL1 promoter in A549 cells (Fig. 5k). Moreover, both the overexpression of LINC00336 and the knockdown of LINC00336 did not change LSH expression levels at the mRNA level (Supplementary Fig. 5g-i). Taken together, these data indicate a regulatory role of LSH in ELAVL1 expression through the p53 signaling pathway.

ELAVL1 regulates the half-life of target RNAs as an RNA-binding protein [42]. To determine whether the RNA stability of LINC00336 is affected by ELAVL1, we treated A549 cells with actinomycin D (Act D) for 0, 1, 2, 4, and 8 h, and measured LINC00336 RNA levels. We found that the half-life of LINC00336 greatly increased after the overexpression of ELAVL1 in A549 cells (Fig. 5l, m) and decreased after the knockdown of ELAVL1 in PC9 cells (Fig. 5n, o). This result suggests that ELAVL1 directly promotes the RNA stability of LINC00336. Finally, we further analyzed the correlation between ELAVL1 and LINC00336 in lung cancer. A strong correlation between ELAVL1 and LINC00336 was found in lung ADC and SCC (Fig. 5p, q). In addition, a strong correlation between LSH and ELAVL1 was found in lung ADC and SCC (Fig. 5r, s).

### LINC00336 regulates CBS expression by competing for MIR6852

**Fig. 6:**
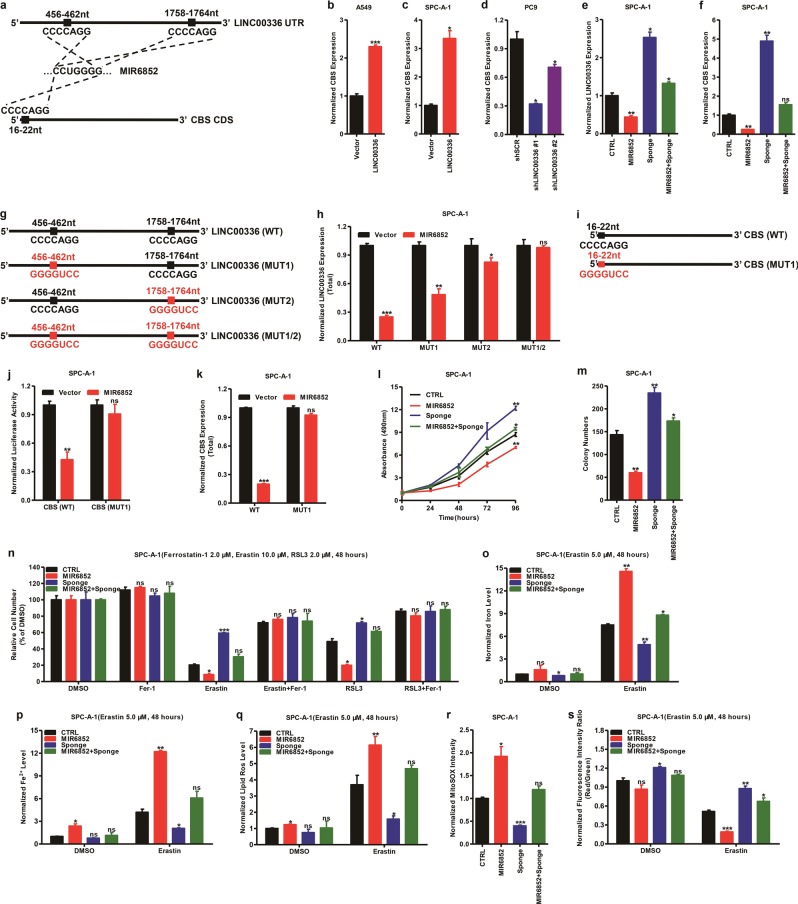
LINC00336 affected the CBS mRNA level by interacting with MIR6852. **a** Predicted MIR6852 binding sites in LINC00336 and CBS. **b**, **c** The mRNA level of CBS increased in A549 (**b**) and SPC-A-1 cells (**c**) stably overexpressing LINC00336. **d** The mRNA level of CBS decreased after the knockdown of LINC00336 in PC9 cells. **e**, **f** The RNA level of CBS (**e**) and LINC00336 (**f**) was detected in stably overexpressing MIR6852 or sponge cells. **g** Scheme of wild-type LINC00336 and of mutants. **h** Detection of total LINC00336 expression levels in overexpressed WT or mutants LINC00336 and MIR6852 in SPC-A-1 cells. **i** Scheme of wild-type CBS and of mutants. **j** The luciferase assay shows that MIR6852 decreased wild-type CBS mRNA stability but not mutant. **k** Detection of total CBS mRNA in overexpressed wild-type or mutant CBS and MIR6852 cells. **l** The MTT assay was used to assess cell viability in stably overexpressed MIR6852 or sponge cells. **m** Growth in the plate was measured using a colony-formation assay of stably overexpressing MIR6852 or sponge cells. **n** MTT assays were used to analyze responses of cells stably overexpressing MIR6852 or sponge to ferrostatin (2.0 μM), erastin (10.0 μM), and RSL3 (2.0 μM). **o**–**s** Level of total iron (**o**), ferrous iron (**p**), lipid ROS (**q**), mitochondrial superoxide (**r**), and mitochondrial membrane potential (**s**) were analyzed in SPC-A-1 cells stably overexpressing MIR6852 or sponge. Data are shown as the mean ± SEM; *n* ≥ 3 independent experiments, two-tailed Student’s *t*-test: ns nonsignificant (*p* > 0.05), *P < 0.05, **P < 0.01, ***P < 0.001

Interactions between lncRNAs and miRNAs, which are important classes of noncoding RNAs in eukaryotes, provide an additional layer of control in gene regulation [15]. Using miRPathDB Microinspector software (https://mpd.bioinf.uni-sb.de), we found a set of miRNAs that putatively bind to LINC00336 (Supplementary Tab. 1). Among these miRNA candidates, we found that MIR6852 directly binds to LINC00336 and cystathionine-β-synthase (CBS), which is involved in ferroptosis as a marker of transsulfuration pathway activity [36, 43] (Fig. 6a and Supplementary Tab. 2). We first addressed the relationship between LINC00336 and CBS. The overexpression of LINC00336 significantly enhanced CBS expression in A549 and SPC-A-1 cells (Fig. 6b, c), whereas the knockdown of LINC00336 significantly inhibited CBS expression in PC9 cells (Fig. 6d). Subsequently, we found that LINC00336 enhanced CBS mRNA levels in transplanted tumors from nude mice (Supplementary Fig. 6a-c), suggesting that LINC00336 might promote CBS expression.

Next, we found that the overexpression of MIR6852 significantly inhibited LINC00336 and CBS expression, whereas a sponge of MIR6852 increased LINC00336 and CBS expression (Fig. 6e, f). We then clarified the relationship between LINC00336 and MIR6852. We found a significant decrease in LINC00336 expression following the transfection of MIR6852 and wild-type LINC00336, but not from mutant LINC00336. The 3′-MIR6852 binding site of LINC00336 presents higher levels of binding activity than the 5′-MIR6852 binding site (Fig. 6g, h). Taken together, these findings indicate that LINC00336 may use sponge MIR6852 as a ceRNA.

Furthermore, dual-luciferase assays show a significant decrease in luciferase activities following the transfection of MIR6852 and the wild-type CBS coding sequence (CDS) region but not for the mutant CBS CDS region of SPC-A-1 cells (Fig. 6i, j). Finally, we found that MIR6852 decreased CBS expression following wild-type CBS but not for mutant CBS (Fig. 6k), indicating that CBS is directly targeted by MIR6852.

To uncover the physiological role of MIR6852 in lung cancer, we first examined the expression levels of MIR6852 in ADC cell lines. MIR6852 expression levels in SPC-A-1 cells were higher than those observed in other ADC cell lines, and this might show that MIR6852 has important functions in SPC-A-1 (Supplementary Fig. 6d). Thus, we introduced MIR6852 and the MIR6852 sponge into SPC-A-1 cells by using lentivirus. The expression levels of MIR6852 had been detected and MIR6852 did not affect the mRNA expression of LSH (Supplementary Fig. 6e, f). We found that MIR6852 overexpression significantly suppressed cellular proliferation, whereas the MIR6852 sponge significantly increased cell proliferation in SPC-A-1 cells (Fig. 6l). In addition, MIR6852 overexpression significantly suppressed colony formation, whereas the MIR6852 sponge significantly increased colony formation (Fig. 6m and Supplementary Fig. 6g). Moreover, we found that the overexpression of MIR6852 increased the erastin- or RSL3-induced growth inhibition of SPC-A-1 cells, and that the MIR6852 sponge decreased erastin- or RSL3-induced growth inhibition (Fig. 6n and Supplementary Fig. 6h, i). The trypan blue staining results show that MIR6852 increased the percentage of dead cells present (Supplementary Fig. 6j). MIR6852 also increased intracellular concentrations of lipid ROS, iron, Fe^2+^, and mitochondrial superoxide, and decreased the mitochondrial membrane potential of SPC-A-1 cells (Fig. 6o–s). Collectively, these data demonstrate that MIR6852 directly binds to LINC0033 and serves as a negative upstream regulator of CBS-mediated ferroptosis inhibition.

## Discussion

ELAVL1 is overexpressed in lung cancer and regulates cell proliferation and survival [44, 45], and miR-519 directly modulates HuR expression [46]. Recently, combined HuR and CXCR4 targeting effectively controls lung cancer metastasis [47]. Here we extend this observation by showing that ELAVL1 inhibits ferroptosis by interacting with LINC00336.

Both lncRNAs and miRNAs have dynamic roles in transcriptional and translational regulation, and are involved in many human diseases, especially in cancer [48]. Interactions between lncRNAs and miRNAs have been reported recently and have attracted interest. For example, CHRF serves as an endogenous “sponge” of miR-489 to regulate Myd88 expression and hypertrophy [49], and miR-21 targets lncRNA GAS5 to suppress GAS5 expression [50]. These discoveries encouraged us to explore the potential roles of miRNAs in binding and regulating lncRNAs. Here we present strong evidence showing that LINC00336 is silenced by MIR6852 in cancer cells. We also examined whether ectopic LSH alters LINC00336 expression, as the overexpression of LSH in lung cancer cells significantly affected LINC00336 expression.

The ferroptotic mode of programmed necrosis has been recently discovered as an apoptosis-independent form of cell death [51]. Ferroptosis is characterized by the iron-dependent lethal accumulation of lipid ROS [33, 37, 38]. Chromatin-modifying enzymes mediate the sensing of intermediary metabolism products to modulate gene regulation and disease progression, including cancer [25–27, 52–54]; however, the link between epigenetic modifications and ferroptosis remains less clear. Here we demonstrate that LINC00336 decreases iron concentrations, lipid ROS, and mitochondrial superoxide, and increases mitochondrial membrane potential consistent with its role in ferroptosis. Moreover, our findings provide evidence that LINC00336 has an important role in lung carcinogenesis. Our findings are the first to suggest that LINC00336 acts as an important inhibitor of ferroptosis in carcinogenesis by interacting with ELAVL1, which acts as a novel regulator of ferroptosis.

**Fig. 7:**
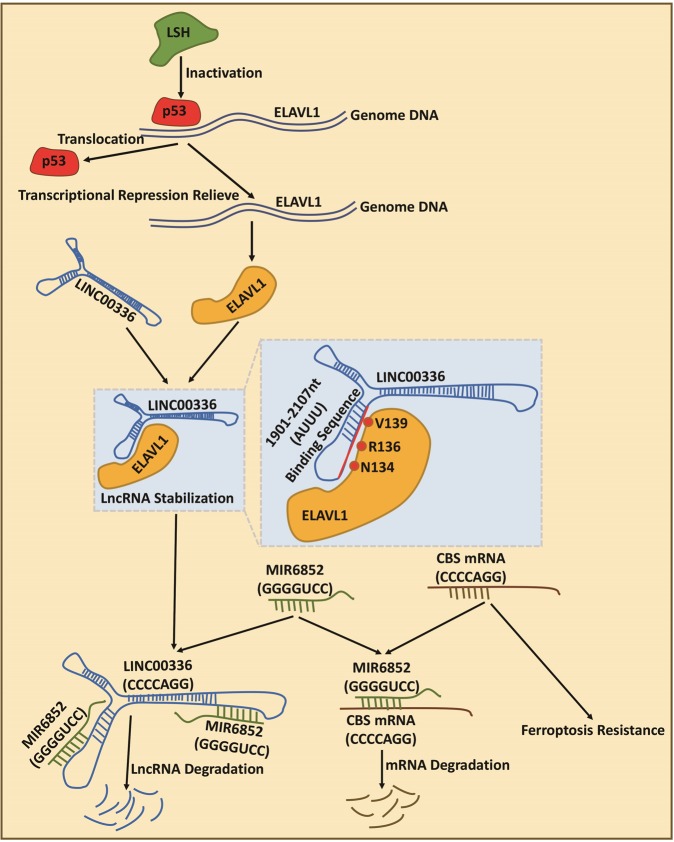
Long noncoding RNA LINC00336 inhibits ferroptosis in lung cancer by functioning as a competing endogenous RNA. LSH induces ELAVL1 expression through the inactivation of p53 and ELAVL1 enhances LINC00336 levels through transcriptional regulation by interacting with LINC00336. Then, LINC00336 absorbs MIR6852 as a ceRNA, which increases the mRNA level of CBS, stimulating cell proliferation, colony formation, and tumor formation, and inhibiting ferroptosis in lung cancer

In summary, our study illustrates that LINC00336 functions as an oncogene to facilitate tumor cell proliferation, inhibit ferroptosis, and induce tumor formation in an ELAVL1-dependent manner. These findings indicate that LINC00336 is a critical molecule for tumor progression and may serve as an effective target of lung cancer therapy (Fig. 7).

## Materials and methods

### Cell culture, chemicals, plasmids, and siRNAs

Lung cancer cell lines A549 (ATCC: CCL-185™) and H358 (ATCC: CRL-5807™) were obtained from American Type Culture Collection. PC9 and SPC-A-1 were obtained from the Cancer Research Institute of Central South University. H358, PC9, and SPC-A-1 cells were cultured in Roswell Park Memorial Institute 1640 Media (RPMI 1640; Gibco, USA) supplemented with 10% fetal bovine serum (FBS). A549 cells were cultured in Dulbecco's Modified Eagle Medium/Nutrient Mixture F-12 (DMEM/F12) 1:1(HyClone), 293T cells were cultured in Dulbecco’s modified Eagle’s medium (Gibco), and the other cell lines were cultured in RPMI 1640 (Gibco). All media were supplemented with 10% (V/V) FBS. All cell lines were maintained at 37 °C with 5% CO_2_. All cell lines tested negative for mycoplasma contamination and were passaged < 10 times after the initial revival from frozen stocks. All cell lines were authenticated before use by short tandem repeat profiling. Erastin was purchased from Selleck (Houston, USA). LSH, LINC00336, ELAVL1, and MIR6825 cDNA clones were purchased from Vigene Biosciences. LSH, LINC00336, ELAVL1 shRNA vectors, and the nonspecific target control (GV248) were purchased from GeneChem (www.genechem.com.cn) (Shanghai, China). Sequences for shRNAs involved are listed in Supplementary Table S3. The transfection of plasmids was performed using Lipofectamine® 2000, according to the manufacturer’s protocol, and stably expressing colonies were selected using 2 mg/ml puromycin.

### Cell proliferation and colony-formation assays

The cell proliferation assay was performed with a CellTiter 96 AQueous One Solution Cell Proliferation Assay (MTS, 3-(4,5-dimethylthiazol-2-yl)-5-(3-carboxymethoxyphenyl)-2-(4-sulfophenyl)-2H-tetrazolium) as per the manufacturer’s protocol. First, 200 cells were cultured in a 96-well plate. The OD450 was measured 1 h after adding MTS. For the cell colony-formation assay, ~200 cells were seeded into the wells of 6-well plates and cultured in media. After 2 weeks, cells were treated with methanol and stained with 0.1% crystal violet. The number of visible colonies was counted using ImageJ software and colonies of > 0.05 mm in diameter were scored.

### Trypan blue staining assays

After 48 h of erastin treatment, cells were collected by trypsinization, centrifuged, and resuspended in phosphate-buffered saline (PBS). Cells were diluted to 1:100 in 0.25% trypan blue solution (Invitrogen) and were counted in a hemocytometer to assess the number of dead blue cells from the total number of cells counted.

### Mitochondrial membrane potential measurements

To measure the mitochondrial membrane potential by using Mitochondrial Membrane Potential Kit MAK-159 (Sigma Aldrich), 5 × 10^4^ cells were seeded in 90 µl, in black 96-well plates with clear bases in phenol red-free medium and treated the next day by adding dimethyl sulfoxide (DMSO) or 10 µM erastin. After 48 h of incubation, 50 µl of JC-10 dye in buffer A (1/100) was added to the cells and incubated for 60 min, and then 50 µl of buffer B was added. Plates were read on a microplate reader. We monitored fluorescence intensity levels (*λ*_ex_ = 490/*λ*_em_ = 525 nm) and (*λ*_ex_ = 540/*λ*_em_ = 590 nm) for ratio analysis. The ratio of red/green fluorescence intensity was used to determine mitochondrial membrane potential.

### Mitochondrial superoxide measurements

Mitochondrial superoxide production in lung cancer cells was measured using MitoSOX™ Red Mitochondrial Superoxide Indicator for live-cell imaging (Invitrogen), a fluorescent probe specifically designed for mitochondrial superoxide. We dissolved 50 μg of MitoSOX mitochondrial superoxide indicator in 13 μl of DMSO to form a 5 mM MitoSOX reagent stock solution. We then diluted 5 mM MitoSOX reagent stock solution in Hank’s buffered salt solution/Ca/Mg buffer to create a 5 μM MitoSOX reagent working solution. We the applied 2.0 ml of 5 μM MitoSOX reagent working solution to cover cells adhering to coverslips. Cells were then incubated for 30 min at 37 °C in dark conditions. The cells were the gently rinsed three times with PBS buffer. Cellular fluorescence was examined under an inverted fluorescence microscope (Olympus) at an excitation/emission value of 510/580 nm. Fluorescence intensity was quantified with ImageJ software (NIH). The results are expressed as relative fluorescence intensity levels normalized to controls.

### Lipid ROS assays

For lipid ROS, cells were treated as indicated, and then trypsinized and resuspended in medium plus 10% FBS. Then, 10 µM of C11-BODIPY (Thermo Fisher, Cat #D3861) was added and the samples were incubated for 30 min at 37 °C, 5% CO_2_, and protected from light. Excess C11-BODIPY was removed by washing the cells with PBS twice. The fluorescence of C11-BODIPY581 = 591 was measured by the simultaneous acquisition of green (484/510 nm) and red signals (581/610 nm) using a flow cytometer.

### Iron assays

For the iron assay, we used an Iron Assay Kit (Sigma Aldrich) to measure Fe^2+^ or total iron in each cell line. First, 2 × 10^6^ of cells was rapidly homogenized in 4–10 volumes of Iron Assay buffer. Samples were centrifuged at 13,000 × *g* for 10 min at 4 °C to remove insoluble material. To measure ferrous iron, 5 µl of iron assay buffer was added to each well. To measure ferric iron, two sets of wells were set up. Then, 5 µl of assay buffer was added to the samples in one set of wells and 5 µl of Iron Reducer was added to the other set of wells. To measure total iron, 5 µl of Iron Reducer was added to each sample well to reduce Fe^3+^ to Fe^2+^. Samples were mixed well using a horizontal shaker or by pipetting and the reactions were incubated for 30 min at room temperature in dark conditions. Then, 100 µl of Iron Probe was added to each well containing standard or test samples. Samples were mixed well using a horizontal shaker or by pipetting and the reactions were incubated for 60 min at room temperature in dark conditions. Finally, the absorbance was measured at 593 nm (A593).

### RT-qPCR assays

Total RNA was isolated using Trizol reagent and assessed using Nanodrop. The primers used are listed in Supplementary Table S4. Real-time PCR was performed using detection system ABI 7500 with FastStart Universal SYBR Green Master (ROX). All gene expression levels were normalized by subtracting the Ct of the ACTB or U6 mRNA level.

### RNA stability analysis

Overexpression or knockdown cells were treated with Act D (50 mg/ml) for 0, 1, 2, 4, or 8 h followed by the extraction of RNA with the Trizol reagent. RT-qPCR and relative RNA analyses were performed using the 2^−ΔΔCt^ method and mRNA levels were calibrated to the 0 h time point.

### Immunoblotting

Cells were collected, washed with PBS, and lysed in IP lysis buffer with protease inhibitor cocktail. Cell lysates were separated on an SDS-polyacrylamide gel, transferred to a polyvinylidene fluoride membrane, and immunoblotted using the following primary antibodies. Protein detection was performed using mouse monoclonal anti-β-actin (Sigma Aldrich, A5441), mouse monoclonal anti-Lsh (Santa Cruz, sc-46665), mouse monoclonal anti-HuR (Santa Cruz, sc-71290), and mouse monoclonal anti-OctA (Santa Cruz, sc-166355).

### RIP assays

First, 4 × 10^7^ cells were crosslinked for 10 min at 37 °C using 0.3% formaldehyde in the medium; this was followed by neutralization with 125 mM glycine at room temperature for 5 min. Cells were washed with cold 1 × PBS twice and then lysed in RIPA buffer (50 mM Tris pH 7.4, 150 mM NaCl, 1 mM EDTA, 0.1% SDS, 1% NP-40, 0.5% sodium deoxycholate, and 0.5 mM dithiothreitol with RNase inhibitor and protease inhibitor cocktail) followed by the mechanical shearing of chromatin using a Dounce homogenizer for 15–20 strokes. Immunoprecipitation was performed by incubating protein A/G magnetic beads with precleared lysates. Lysates were incubated with 2.0 μg of mouse monoclonal anti-HuR/ELAVL1 (Santa Cruz, sc-71290) or 2.0 μg of mouse monoclonal anti-OctA (Santa Cruz, sc-166355) with an equivalent amount of normal mouse IgG at 4 °C for 16 h. The RNA/antibody complex was then precipitated by incubation with protein A/G magnetic beads. After three rounds of rinsing with ice-cold 1 × PBS, the RNA samples were extracted with Trizol reagent and detected by RT-qPCR.

### ChIP assays

At room temperature, 5 × 10^6^ cells were fixed with 1% formaldehyde (Sigma) for 10 min. Fixation was stopped with the addition of a 1/10 volume of 1.25 M glycine and with incubation for 5 min at room temperature. The sonication step was performed in a Qsonica sonicator (for 5 min alternating 20 s on and 20 s off) and 200 μg of the protein–chromatin complex was used for each round of IP. The antibody–protein complex was captured with preblocked Pierce Protein A/G Magnetic Beads. ChIP DNA was analyzed by qPCR with FastStart Universal SYBR Green Master (ROX) in an ABI 7500 using the following oligonucleotides: F: 5′-GCTGAGCCTTCGGGAACTGGCTTGC-3′ and R: 5′-CCGTTGGGCAGGAGCTGACGTCACC-3′. The following antibodies were used: 4.0 μg of mouse monoclonal anti-p53 (Santa Cruz, sc-126) and 4.0 μg of normal mouse IgG (Santa Cruz, sc-2025).

### Biotinylated RNA pull-down assays

Biotin-labeled RNAs were transcribed in vitro using a TranscriptAid T7 High Yield Transcription Kit. Cells were cultured on 10 cm plates until 80% confluent. Subsequently, 4 × 10^7^ cells were washed with ice-cold 1 × PBS. Then, 1 ml of IP lysis buffer supplemented with 1 mM phenylmethylsulfonyl fluoride was added to the plates, which were then incubated on ice for 5 min. Lysates were centrifuged for 20 min at 15,000 × *g* at 4 °C. The supernatant was transferred to a new microcentrifuge tube. The RNA pull-down assay was performed using a Magnetic RNA-Protein Pull-Down Kit according to the manufacturer’s instructions. First, the biotin-labeled RNA was bound to the beads for protein binding. Cell protein lysate was then added to the RNA-bound beads for 16 h of IP. The beads were then washed three times and boiled in SDS buffer; the pull-down complexes were analyzed using an immunoblotting technique.

### Dual-luciferase assays

To test how MIR6852 influences the activity of CBS mRNA, we used a Dual-Luciferase Reporter Assay System (Promega, E1910 and E1960). The growth medium was removed from the cultured cells and the surface of the culture vessel was washed with PBS. Into each culture well, the minimum volume of 1 × Passive Lysis Buffer (1 × PLB) required to completely cover the cell monolayer was added. The culture plates were placed on a rocking platform or orbital shaker with gentle rocking/shaking, to ensure the complete and even coverage of the cell monolayer with 1 × PLB. The culture plates were rocked at room temperature for 15 min. The lysate was transferred to a tube and reporter assays were directly performed in the wells of the culture plate. The lysate samples were cleared for 30 s by centrifugation at rapid speeds in a refrigerated microcentrifuge. The cleared lysates were then transferred to a new tube prior to reporter enzyme analyses. Next, 100 μl of LAR II was added to the appropriate number of luminometer tubes to complete the desired number of DLR assays. Up to 20 μl of cell lysate was then carefully transferred into the luminometer tube containing LAR II and was mixed by pipetting two or three times. Next, 100 μl of Stop & Glo® Reagent was added and briefly vortexed to mix. The sample was then placed in the luminometer and read.

### Statistical analysis

All statistical analyses were performed using the Graphpad Prism 8.0 software package and SPSS 22.0 statistical software package (Abbott Laboratories, USA) for Windows. The data are presented as mean ± SEM from multiple individual experiments each performed in triplicate and each experiment was repeated at least three times. Student’s *t*-test (two-tailed) was applied to compare difference between two groups: ns is nonsignificant (*P* > 0.05), *P < 0.05, **P < 0.01, ***P < 0.001. The relationship between LSH, LINC00336, and ELAVL1 expression levels was determined by using the Spearman’s correlation coefficient (*r*), where the *p*-value reflects the result of a *t*-test with the null hypothesis that the correlation between the variables is equal to zero. Survival analyses were conducted considering the time from diagnosis to the date of the event (death, relapse, or last follow-up). Overall and disease-free survivals were estimated using the Kaplan–Meier method and compared using a log-rank test; the number of patients is presented as “*n*” in the text, where the *p*-value reflects whether the difference between the survival curves is significant.

## Supplementary information

Supplementary Figure 1

Supplementary Figure 2

Supplementary Figure 3

Supplementary Figure 4

Supplementary Figure 5

Supplementary Figure 6

Supplementary figure legends

Supplementary tables
