# Supplementary figures and images for: Long noncoding RNA LINC00336 inhibits ferroptosis in lung cancer by functioning as a competing endogenous RNA
Source: Cell Death Differ. 2019 Feb 20;26(11):2329–43. doi: 10.1038/s41418-019-0304-y (PMC6889193; doi:10.1038/s41418-019-0304-y)

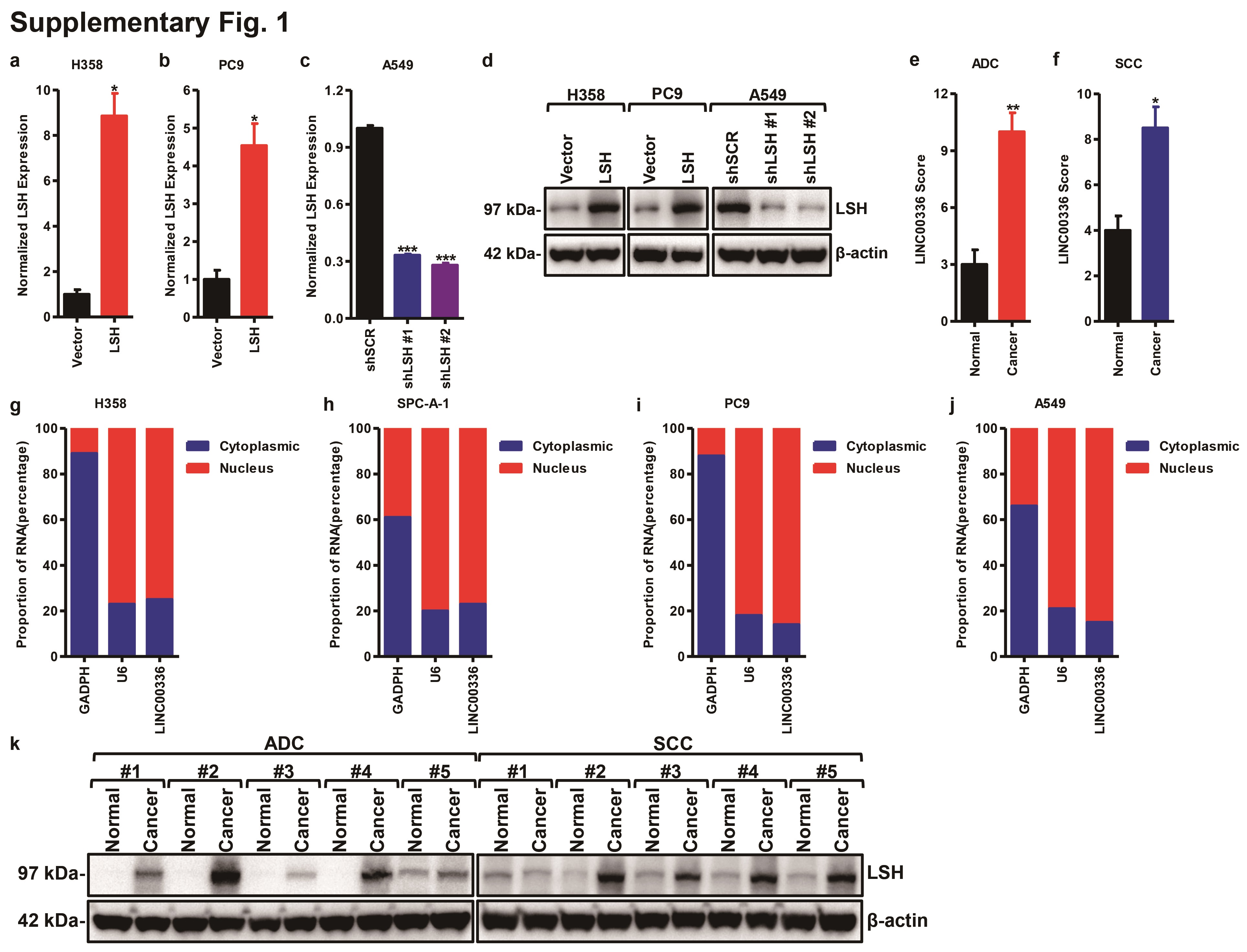

Supplement: Supplementary file 1 — Supplementary Figure 1 [file 41418_2019_304_MOESM1_ESM.jpg]

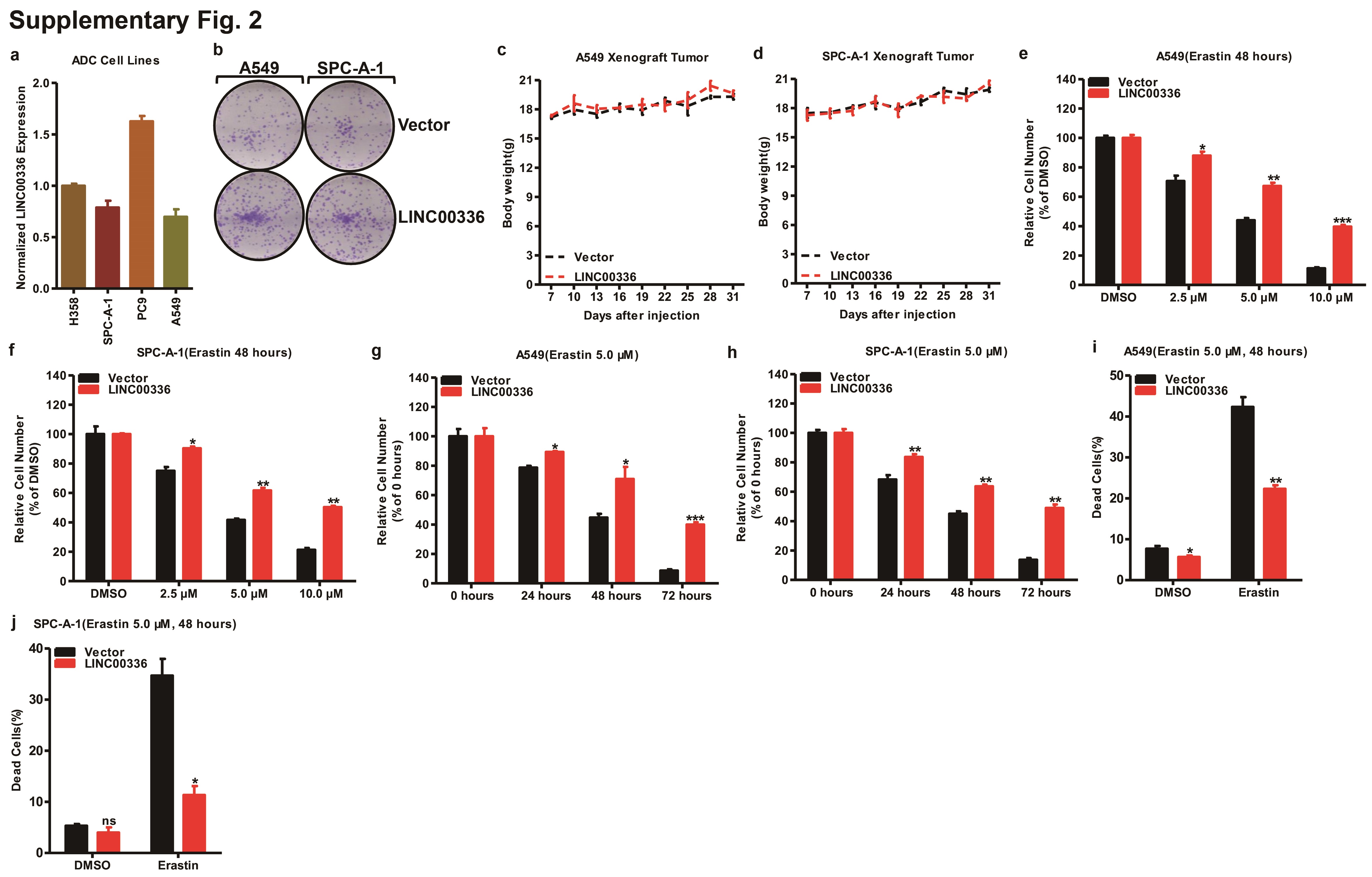

Supplement: Supplementary file 2 — Supplementary Figure 2 [file 41418_2019_304_MOESM2_ESM.jpg]

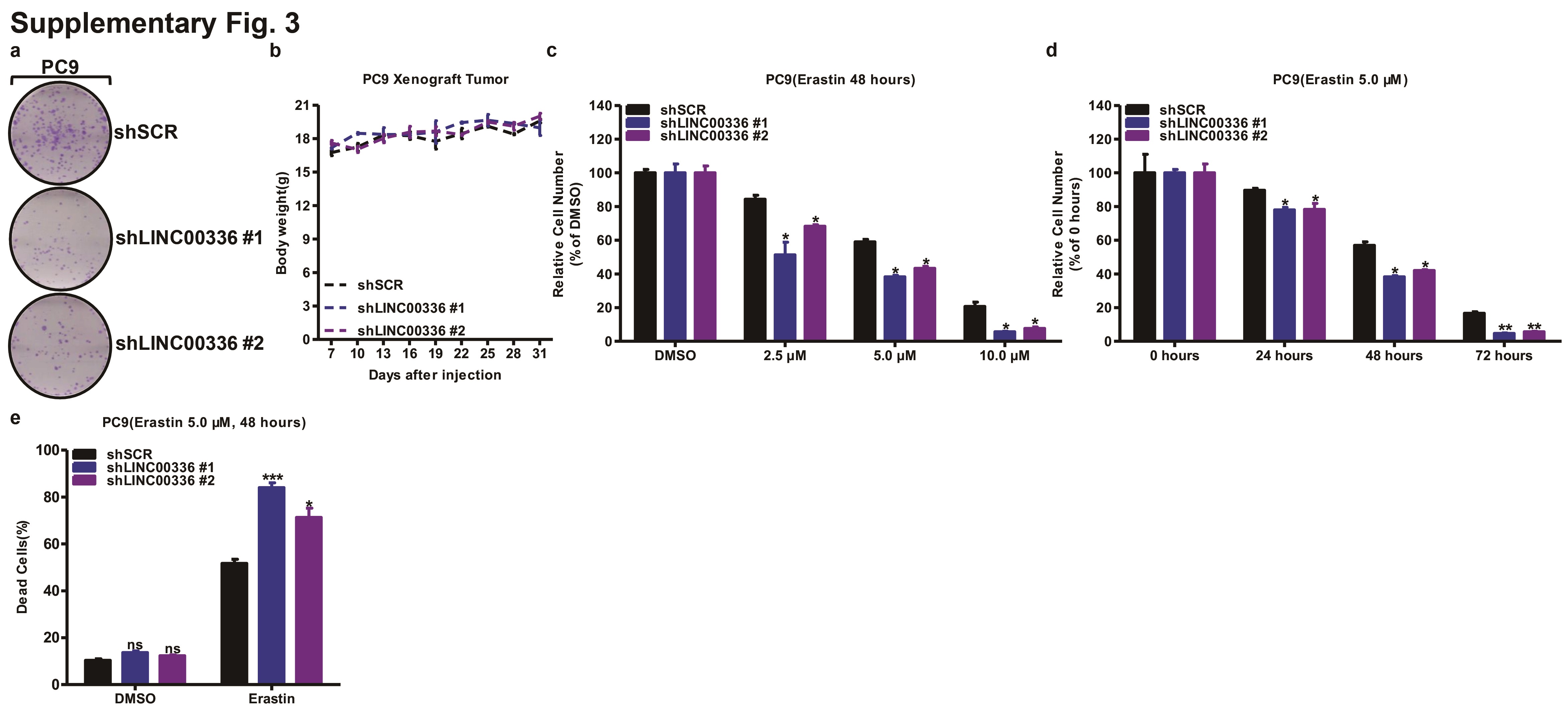

Supplement: Supplementary file 3 — Supplementary Figure 3 [file 41418_2019_304_MOESM3_ESM.jpg]

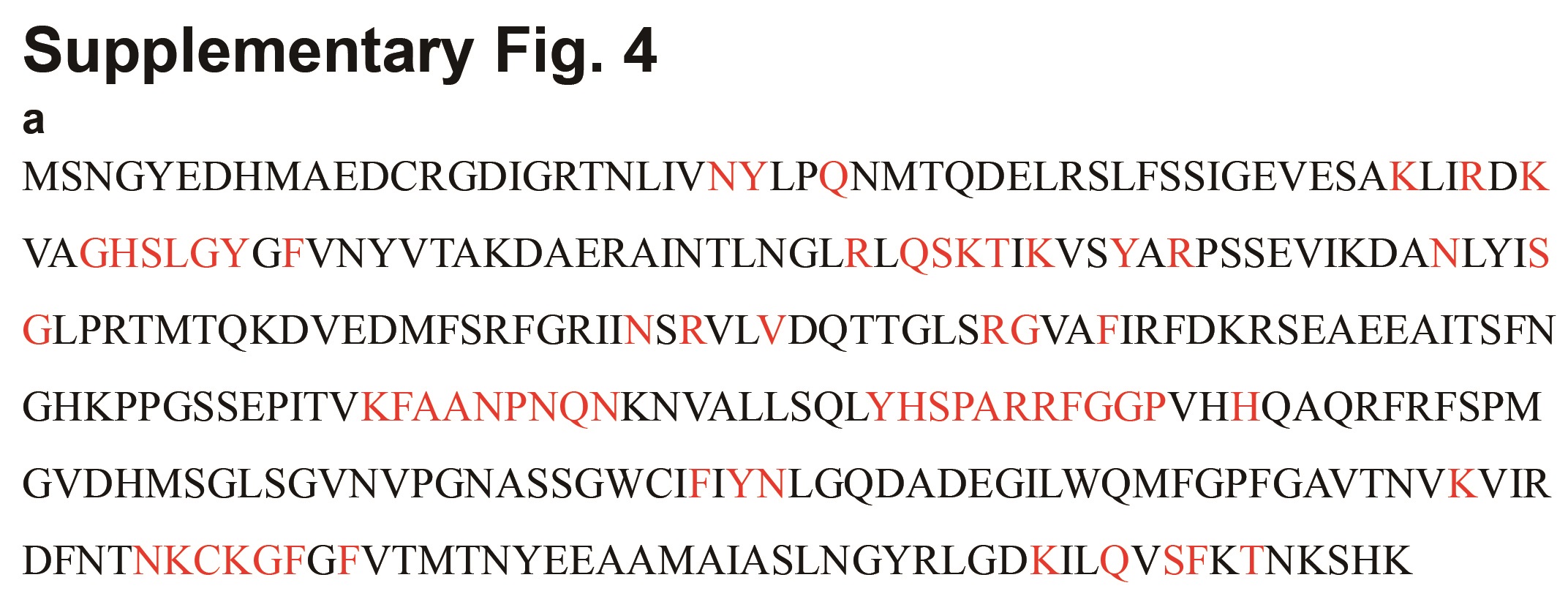

Supplement: Supplementary file 4 — Supplementary Figure 4 [file 41418_2019_304_MOESM4_ESM.jpg]

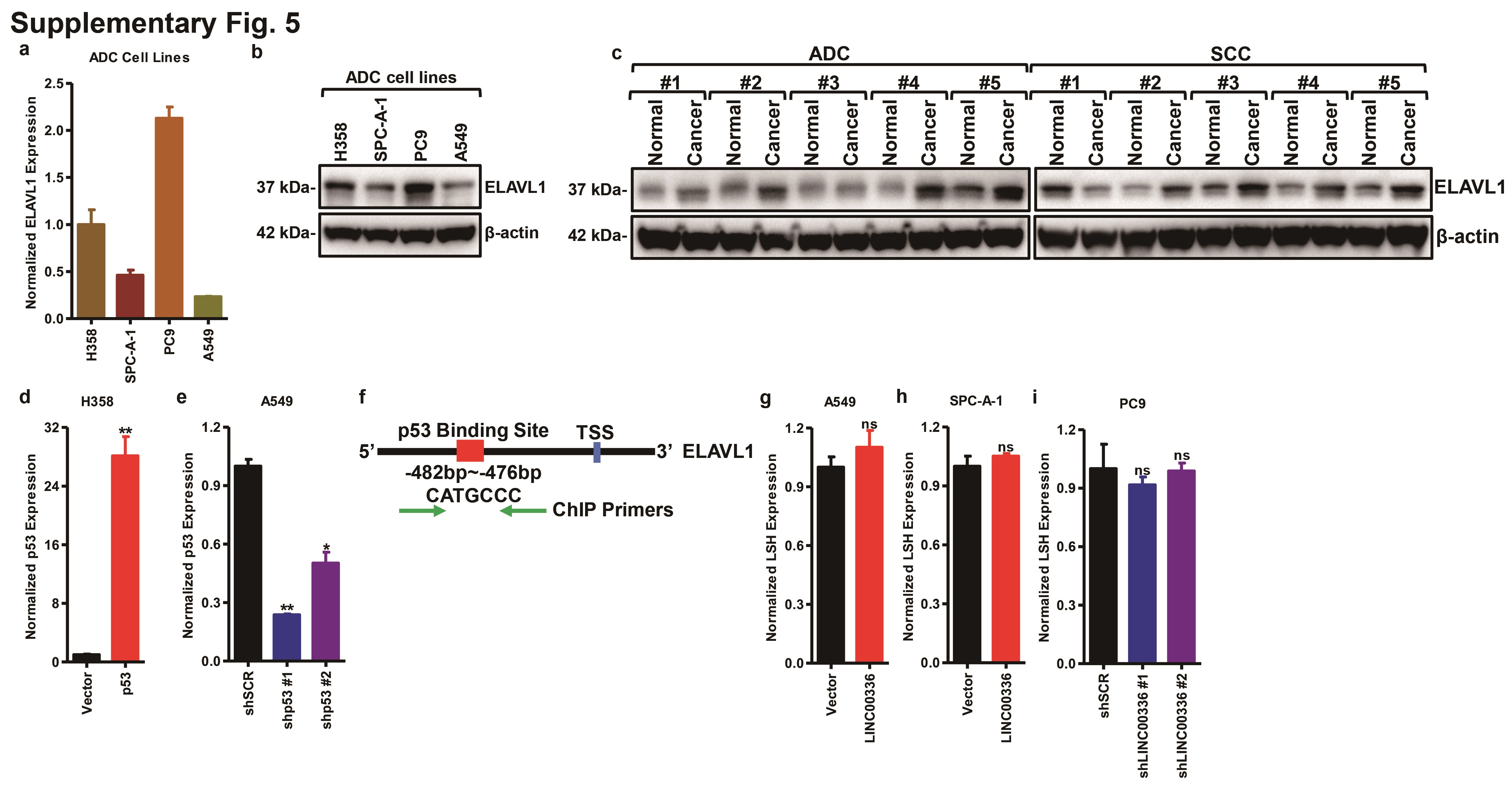

Supplement: Supplementary file 5 — Supplementary Figure 5 [file 41418_2019_304_MOESM5_ESM.jpg]

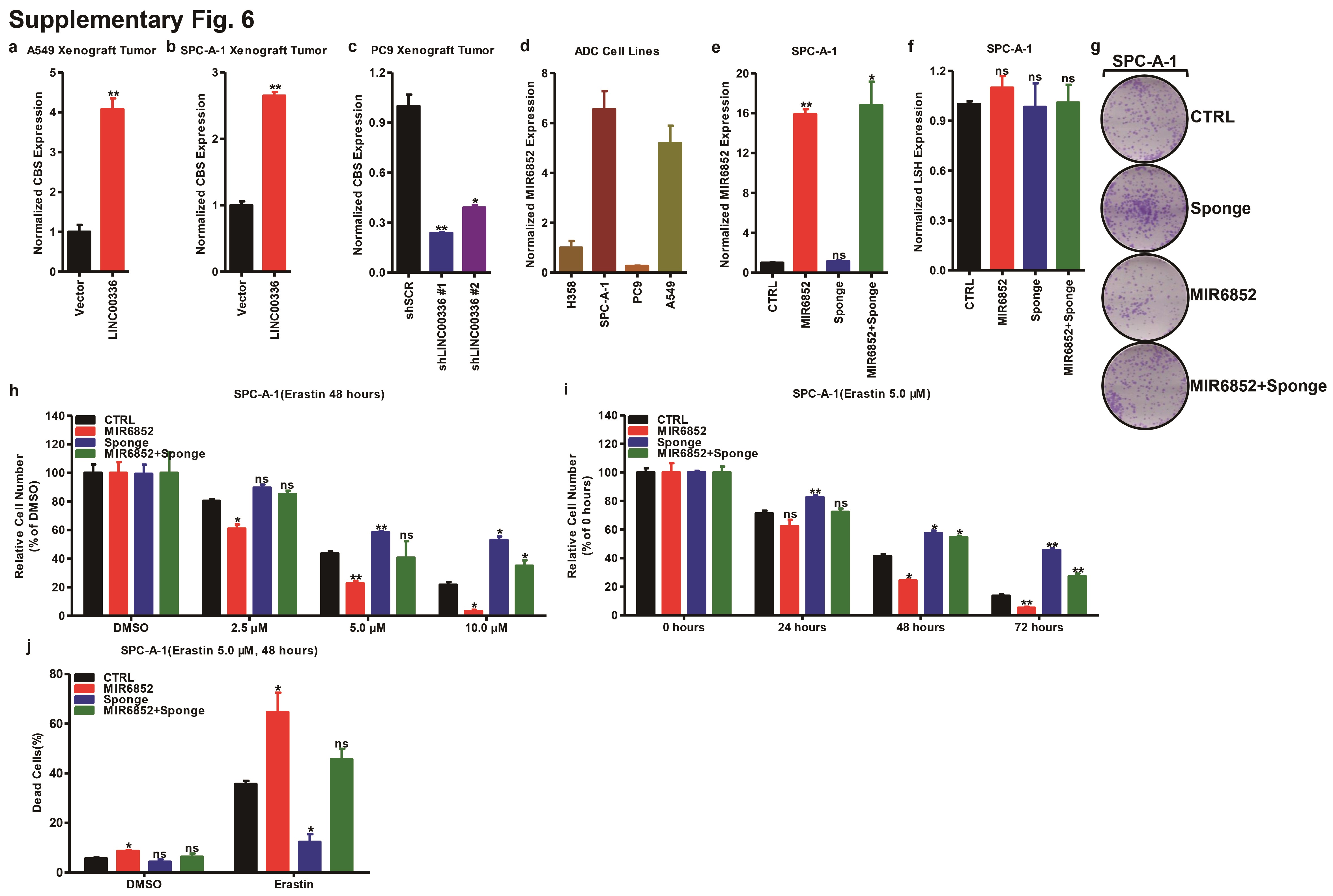

Supplement: Supplementary file 6 — Supplementary Figure 6 [file 41418_2019_304_MOESM6_ESM.jpg]
