# Supplementary material for: Long noncoding RNA LINC00336 inhibits ferroptosis in lung cancer by functioning as a competing endogenous RNA
Source: Cell Death Differ. 2019 Feb 20;26(11):2329–43. doi: 10.1038/s41418-019-0304-y (PMC6889193; doi:10.1038/s41418-019-0304-y)
Supplement: Supplementary file 7 — Supplementary figure legends [file 41418_2019_304_MOESM7_ESM.docx]

**Long noncoding RNA LINC00336 inhibits ferroptosis in lung cancer by functioning as a competing endogenous RNA**

Min Wang ^1,2,3^, Chao Mao ^1,3^, Lianlian Ouyang ^1,3,4^, Yating Liu ^1,3^, Weiwei Lai ^1,3^, Na Liu ^1,3^, Ying Shi ^1,3^, Ling Chen ^1,3^, Desheng Xiao ^5^, Fenglei Yu ^6^,Xiang Wang ^6^, Hu Zhou ^7^, Ya Cao ^1,3^, Shuang Liu ^4^, Qin Yan ^8^, Bin Zhang ^2^, * and Yongguang Tao ^1,3,6^, *

^1^ Key Laboratory of Carcinogenesis and Cancer Invasion, Ministry of Education, Xiangya Hospital, Central South University, Hunan, 410078 China

^2^ Department of Histology and Embryology, School of Basic Medicine, Central South University, Changsha, Hunan, 410013 China

^3^ Key Laboratory of Carcinogenesis of Ministry of Health, Cancer Research Institute, Central South University, Changsha, Hunan, 410078 China

^4^ Institute of Medical Sciences, Xiangya Hospital, Central South University, Changsha, Hunan, 410008 China

^5^ Department of Pathology, Xiangya Hospital, Central South University, Changsha, Hunan, 410008 China

^6^ Department of Thoracic Surgery, Second Xiangya Hospital, Central South University, Changsha, 410011 China

^7^ Shanghai Institute of Material Medica, Chinese Academy of Sciences (CAS), 555 Zu Chongzhi Road, Zhangjiang Hi-Tech Park, Shanghai, 201203, China

^8^ Department of Pathology, Yale School of Medicine, New Haven, CT 06520, USA

**Supplementary Figure Legends**

**Supplementary Fig. 1** Detection of LSH overexpression or knockdown efficiency, LSH and LINC00336 expression levels and LINC00336 subcellular localization. **a-c** The overexpression of LSH in H358 (**a**) and PC9 (**b**) cells and the knockdown of LSH in A549 (**c**) cells for two separate shRNA sequences was detected by qRT-PCR. **d** The overexpression of LSH in H358 and PC9 cells and the knockdown of LSH in A549 cells was detected by western blot. **e, f** ISH scores of LINC00336 expression levels in lung cancer versus normal tissues. **g-j** The subcellular localization of LINC00336 in a panel of lung adenocarcinoma cells. **k** Western blot results show an increased expression of LSH protein levels in 10 paired lung cancer relative to adjacent normal lung tissue samples in ADC and SCC. Data are shown as the mean± s.e.m.; n≥3 independent experiments, two-tailed Student’s t-test: ns is non-significant (p > 0.05), * P<0.05, ** P<0.01, *** P<0.001.

**Supplementary Fig. 2** Images of a colony formation assay in overexpressing LINC00336 cell lines and weights of mice after the injection of overexpressing LINC00336 cells. **a** qPCR analysis was used to detect expression levels of LINC00336 in a panel of lung adenocarcinoma cells. **b** Representative images of a colony formation assay of A549 and SPC-A-1 cells stably overexpressing LINC00336. **c, d** Nude mice after the injection of A549 (**c**) and SPC-A-1 (**d**) cells stably expressing control or LINC00336 overexpression vectors for 30 days; the weights of the mice were recorded at the indicated times (n=6). **e**-**h** MTT assay showing that the overexpression of LINC00336 in A549 and SPC-A-1 cells resists erastin induced ferroptosis in a dose-dependent (**e, f**) and time-dependent (**g, h**) manner. **i, j** Trypan blue staining results showing the percentage of dead cells of A549 (**i**) and SPC-A-1 (**j**) and the overexpression LINC00336 cells treated with erastin. Data are shown as the mean± s.e.m.; n≥3 independent experiments, two-tailed Student’s t-test: ns is non-significant (p > 0.05), * P<0.05, ** P<0.01, *** P<0.001.

**Supplementary Fig. 3** Images of a colony formation assay of knockdown LINC00336 cell lines and the weights of mice after the injection of knockdown LINC00336 cell lines. **a** Representative images of the colony formation assay of PC9 cells after the knockdown of LINC00336. **b** Nude mice after the injection of PC9 cell knockdown LINC00336 with body weights recorded at the indicated time points (n=6). **c, d** MTT assay showing that the knockdown of LINC00336 in PC9 cells promotes erastin-induced ferroptosis in a dose-dependent (**c**) and time-dependent (**d**) manner. **e** Trypan blue staining results showing the percentage of dead PC9 knockdown LINC00336 cells treated with erastin. Data are shown as the mean± s.e.m.; n≥3 independent experiments, two-tailed Student’s t-test: ns is non-significant (p > 0.05), * P<0.05, ** P<0.01, *** P<0.001.

**Supplementary Fig. 4** RNA-binding sites of ELAVL1. **a** RNA-binding sites of ELAVL1 are shown in red.

**Supplementary Fig. 5** ELAVL1 expression levels in lung adenocarcinoma cells, lung-tissue samples and LSH expression levels observed in LINC00336 overexpression or knockdown cell lines. **a, b** qPCR (**a**) and western blot (**b**) analyses were used to detect the expression levels of ELAVL1 in a panel of lung adenocarcinoma cells. **c** Western blot results show an increased expression of ELAVL1 in 10 paired lung-cancer and corresponding normal lung-tissue samples in lung ADC and SCC. **d, e** The overexpression of p53 in H358 cells (**d**) and the knockdown of p53 in A549 cells (**e**) using two separate shRNA sequences were detected by qRT-PCR. **f** The predicted p53 binding site in the ELAVL1 promoter region. **g-i** mRNA levels of LSH undergo no significantly changes when stably overexpressing LINC00336 in A549 (**g**) and SPC-A-1 (**h**) or during the knockdown of LINC00336 in PC9 (**i**). Data are shown as the mean± s.e.m.; n≥3 independent experiments, two-tailed Student’s t-test: ns is non-significant (p > 0.05), * P<0.05, ** P<0.01, *** P<0.001.

**Supplementary Fig. 6** MIR6852 expression level in lung adenocarcinoma cell lines. Images of the colony formation assay of overexpressed MIR6852 cell lines and CBS mRNA levels in xenograft tumors. **a, b** mRNA levels of CBS are enhanced in A549 (**a**) and SPC-A-1 (**b**) stably overexpressed LINC00336 xenograft tumors. **c** mRNA levels of CBS decreased after the knockdown of LINC00336 in PC9 xenograft tumors. **d** qPCR analyses were used to detect expression levels of MIR6852 in a panel of lung adenocarcinoma cells. **e, f** The expression level of MIR6852 (**e**) or LSH (**f**) was detected with qRT-PCR in SPC-A-1 cell overexpressed MIR6852 or sponge. **g** Representative images of colony formation assays of cells stably overexpressed in MIR6852 or MIR6852 sponge. **h, i** MTT assay showing overexpressed MIR6852 or sponge in SPC-A-1 cells affected by erastin-induced ferroptosis in a dose-dependent (**h**) and time-dependent (**i**) manner. **j** Trypan blue staining results showing the percentage of dead cells of overexpressed MIR6852 or sponge in SPC-A-1 cells treated with erastin. Data are shown as the mean± s.e.m.; n≥3 independent experiments, two-tailed Student’s t-test: ns is non-significant (p > 0.05), * P<0.05, ** P<0.01, *** P<0.001.
