# Supplementary material for: Long noncoding RNA LINC00336 inhibits ferroptosis in lung cancer by functioning as a competing endogenous RNA
Source: Cell Death Differ. 2019 Feb 20;26(11):2329–43. doi: 10.1038/s41418-019-0304-y (PMC6889193; doi:10.1038/s41418-019-0304-y)
Supplement: Supplementary file 8 — Supplementary tables [file 41418_2019_304_MOESM8_ESM.docx]

**Long noncoding RNA LINC00336 inhibits ferroptosis in lung cancer by functioning as a competing endogenous RNA**

Min Wang ^1,2,3^, Chao Mao ^1,3^, Lianlian Ouyang ^1,3,4^, Yating Liu ^1,3^, Weiwei Lai ^1,3^, Na Liu ^1,3^, Ying Shi ^1,3^, Ling Chen ^1,3^, Desheng Xiao ^5^, Fenglei Yu ^6^,Xiang Wang ^6^, Hu Zhou ^7^, Ya Cao ^1,3^, Shuang Liu ^4^, Qin Yan ^8^, Bin Zhang ^2^, * and Yongguang Tao ^1,3,6^, *

^1^ Key Laboratory of Carcinogenesis and Cancer Invasion, Ministry of Education, Xiangya Hospital, Central South University, Hunan, 410078 China

^2^ Department of Histology and Embryology, School of Basic Medicine, Central South University, Changsha, Hunan, 410013 China

^3^ Key Laboratory of Carcinogenesis of Ministry of Health, Cancer Research Institute, Central South University, Changsha, Hunan, 410078 China

^4^ Institute of Medical Sciences, Xiangya Hospital, Central South University, Changsha, Hunan, 410008 China

^5^ Department of Pathology, Xiangya Hospital, Central South University, Changsha, Hunan, 410008 China

^6^ Department of Thoracic Surgery, Second Xiangya Hospital, Central South University, Changsha, 410011 China

^7^ Shanghai Institute of Material Medica, Chinese Academy of Sciences (CAS), 555 Zu Chongzhi Road, Zhangjiang Hi-Tech Park, Shanghai, 201203, China

^8^ Department of Pathology, Yale School of Medicine, New Haven, CT 06520, USA

**Supplementary Tables**

**Supplementary Tab. 1** miRNAs targeting LINC00336(miRDB)

| Name | Gene ID | Target LncRNA Sequences |
| --- | --- | --- |
| MIR103 | 100302238 | GGCUAUG |
| MIR337 | 494326 | CAACCUC |
| MIR661 | 724031 | CCCAGGC |
| MIR874 | 100126343 | CAGGGCA |
| MIR1267 | 100302286 | UCAACAG |
| MIR1272 | 100302184 | UCAUCAU |
| MIR1343 | 100616437 | GCUCCCC |
| MIR1914 | 100302137 | GCACAGG |
| MIR3121 | 100423032 | GCAAAGG |
| MIR4254 | 100423028 | CUCCAGG |
| MIR4443 | 100616407 | GCCUCCA |
| MIR4447 | 100616485 | CCCCCAC |
| MIR4492 | 100616376 | CCAGCCC |
| MIR4497 | 100616454 | UCCCGGA |
| MIR4792 | 100616448 | GCUCACC |
| MIR6086 | 102466519 | CAACCUC |
| MIR6852 | 102465513 | CCCCAGG |
| MIR7162 | 102466227 | ACCUCAG |
| MIR8485 | 103504737 | UGUGUGU |

**Supplementary Tab. 2** miRNAs targeting CBS(miRDB)

| Name | Gene ID | Target mRNA Sequences |
| --- | --- | --- |
| MIR198 | 406975 | UCUGGAC |
| MIR483 | 619552 | CCCGUCU |
| MIR548AS | 100847092 | GGGUUUU |
| MIR671 | 768213 | GGCUUCC |
| MIR3918 | 100500851 | GGCCCUG |
| MIR4291 | 100422927 | CUGCUGA |
| MIR4317 | 100422840 | GGCAAUG |
| MIR4729 | 100616204 | AUAAAUG |
| MIR4786 | 100616417 | UGGCUUC |
| MIR5006 | 100847026 | CCUGGCA |
| MIR5010 | 100847046 | ACACAAA |
| MIR5088 | 100847074 | GAGCCCU |
| MIR6785 | 102466911 | CCCUCCC |
| MIR6852 | 102465513 | CCCCAGG |
| MIR6889 | 102466758 | GGCACAG |
| MIR7106 | 102466222 | UCCUCCC |
| MIR7110 | 102465667 | GAGAGAG |
| MIR8070 | 102465870 | AAUCACA |

**Supplementary Tab. 3** shRNA and miRNA sponge target sequences

| Name | Source | Target Sequences |
| --- | --- | --- |
| shLSH #1 | Genechem | AACAAGGCGATAAACAACAAC |
| shLSH #2 | Genechem | AATTGTTTCTTTCTCACTGGA |
| shLINC00336 #1 | Genechem | GAGATGATGAATGGACGGAAA |
| shLINC00336 #2 | Genechem | AACAGAGAGACTGATGGAGAA |
| shELAVL1 #1 | Genechem | TGGATCAGACTACAGGTTT |
| shELAVL1 #2 | Genechem | AGCATTGGTGAAGTTGAAT |
| shp53 #1 | Genechem | CCGGACGATATTGAACAATGGTTCACTGA |
| shp53 #2 | Genechem | CAGCCAAGTCTGTGACTTGCACGTACTCC |
| MIR6852 sponge | Sangon Biotech | CATGTCCTCACAACCCCAGGG |

**Supplementary Tab. 4** RT-PCR primers

| Name | Full Name | Gene ID | Sequences |
| --- | --- | --- | --- |
| LSH | helicase, lymphoid specific | 3070 | F:GATTTTGGATCGAATGCTGCCAG |
|  |  |  | R:ATGGACCCATCAAGCCTGCTGA |
| LINC00336 | long intergenic nonprotein coding RNA 336 | 401253 | F:CCACTGAGCAGAGGTGCTGGC |
|  |  |  | R:GTGCAGTGGCTCACGCCTATAA |
| ELAVL1 | ELAV-like RNA binding protein 1 | 1994 | F:TGTTCTCTCGGTTTGGGCGGAT |
|  |  |  | R:TCTTCTGCCTCCGACCGTTTGT |
| ACTB | actin beta | 60 | F:CACCATTGGCAATGAGCGGTTC |
|  |  |  | R:AGGTCTTTGCGGATGTCCACGT |
| RNU6-1 | U6 small nuclear 1 | 26827 | F:CTCGCTTCGGCAGCACA |
|  |  |  | R:AACGCTTCACGAATTTGCGT |
| GAPDH | glyceraldehyde-3-phosphate dehydrogenase | 2597 | F:GTCTCCTCTGACTTCAACAGCG |
|  |  |  | R:ACCACCCTGTTGCTGTAGCCAA |
| CBS | cystathionine-beta-synthase | 875 | F:CATTGCCAGGAAGCTGAAGGAG |
|  |  |  | R:TTCCACCTCGTAGGTTGTCTGC |
| MIR6852 | microRNA 6852 | 102465513 | F:CCCTGGGGTTCTGAGGACATG |
| p53 | tumor protein p53 | 7157 | F:CCTCAGCATCTTATCCGAGTGG |
|  |  |  | R:TGGATGGTGGTACAGTCAGAGC |
